# Are Selected Cytokines and Epstein–Barr Virus DNA Load Predictors of Hepatological Complications of Epstein–Barr Virus Infection in Children?

**DOI:** 10.3390/jcm12196158

**Published:** 2023-09-24

**Authors:** Justyna Moppert, Krzysztof Domagalski, Sylwia Wrotek, Małgorzata Pawłowska

**Affiliations:** 1 Department of Infectious Diseases and Hepatology, Collegium Medicum in Bydgoszcz, Nicolaus Copernicus University in Torun, 87-100 Toruń, Poland; mpawlowska@cm.umk.pl; 2 Department of Paediatrics, Infectious Diseases and Hepatology, Voivodeship Infectious Observation Hospital in Bydgoszcz, 85-030 Bydgoszcz, Poland; 3 Department of Immunology, Faculty of Biological and Veterinary Sciences, Nicolaus Copernicus University in Torun, 87-100 Toruń, Poland; krydom@umk.pl (K.D.); wrotek@umk.pl (S.W.)

**Keywords:** EBV infection, hepatitis, cytokines, EBV DNA load

## Abstract

The aim of the study was to evaluate tumour necrosis factor-α (TNF-α), interleukin-6 (IL-6), soluble intracellular adhesion molecules 1 (s-ICAM-1) and Epstein–Barr virus (EBV) DNA load levels as predictors of hepatological complications of EBV infection in children. The study group consisted of 54 children aged one to eighteen years, who were hospitalised from 1 December 2018 to 31 December 2020 in the Department of Paediatrics, Infectious Diseases and Hepatology and who had hepatological complications in the course of serologically and molecularly confirmed EBV infection. It was shown that IL-6, TNF-α, and s-ICAM-1 concentrations were the highest in patients with hepatitis and biliary pole damage. Higher EBV DNA viremia positively correlated with increased C-reactive protein (CRP) and TNF-α levels and increased leukocyte, lymphocyte, and monocyte counts. Increases in lymphocyte counts and TNF-α concentrations were observed along with increases in gamma-glutamyl transpeptidase (GGTP) activity. Increased concentrations of IL-6, TNF-α, and s-ICAM-1 may indicate the risk of hepatitis with concomitant biliary pole damage during EBV infection.

## 1. Introduction

In the paediatric population, Epstein–Barr virus (EBV) infection has a very broad symptomatology, ranging from the most commonly observed asymptomatic course to rarely seen acute liver failure with severe cholestasis. Heterophilic anti-EBV antibodies in children between two and five years of age are present in only 50% of children, and in 10–30% of children in the group below two years of age [1]. In laboratory diagnosis, antibodies to the viral capsid antigen (VCA), early antigen (EA), and Epstein–Barr nuclear antigen (EBNA) are most important [2]. However, note that in cases where functional lymphocyte deficiencies are observed, basing the diagnosis on serological tests alone is insufficient. Among immunocompetent patients, the assessment of EBV DNA viral load, especially in cases of primary infection, is generally not performed. However, given the high rate of false-negative serological results in the youngest children, molecular testing may become an important element in differential diagnosis. Viral infections, including infections with EBV, may be one of the factors contributing to endothelium damage and increased leukocyte adhesion. The induction of intracellular adhesion molecules-1 (ICAM-1) secretion and the increased concentration of its soluble form in serum is the result of the action of inflammatory mediators, e.g., tumour necrosis factor-α (TNF-α), interleukin-1(IL-1), interferon γ, and bacterial lipopolysaccharide (LPS), released from inflammation-activated Kupffer cells and damaged hepatocytes [3,4,5,6]. TNF-α is a pro-inflammatory multifunctional cytokine produced mainly by monocytes, macrophages, and T lymphocytes. The effect on immune cells is due to both direct action and synergy with cytokines such as interleukin-6 (IL-6) or IL-1. The enhancement of the inflammatory response and stimulation of acute-phase protein production in the liver by TNF-α results from the stimulation of chemotaxis and phagocytosis of macrophages, monocytes, and neutrophils and an increase in their adhesion to endothelial cells via an increased secretion of ICAM-1 [7,8]. TNF-α-mediated production of acute phase proteins in the liver, including C-reactive protein (CRP), allows plasma CRP concentrations to be utilised to monitor the TNF-α-dependent inflammatory response. IL-6 is a pleiotropic cytokine whose production is induced by multiple factors, i.e., IL-1, TNF-α, interferons, viruses, and bacterial LPS [9]. It is the main initiator of acute-phase protein synthesis, the vast majority of which is produced in the liver. The aim of the study was to evaluate TNF-α, IL-6, s-ICAM-1, and EBV DNA load levels as predictors of hepatological complications of EBV infection in children.

## 2. Materials and Methods

The study included 68 immunocompetent children, comprising 36 girls and 32 boys aged between 1 and 18 years, hospitalised from 1 December 2018 to 31 December 2020 in the Department of Paediatrics, Infectious Diseases and Hepatology with serologically and molecularly confirmed EBV infection. The study group consisted of 54 children with hepatological complications. Two subgroups were identified: with increased alanine aminotransferase (ALT) and gamma-glutamyl transpeptidase (GGTP) activity, to which 33 patients were classified (Group IA), and a 21-person subgroup of patients with increased ALT activity and normal GGTP (Group IB). The control group consisted of 14 EBV-infected children with normal ALT and GGTP activity (Group II). The comparison group consisted of 13 healthy children in whom EBV genetic material was excluded by molecular testing (Group III).

This study was approved by the scientific and ethical committees of Nicolaus Copernicus University in Torun, Ludwik Rydygier Collegium Medicum in Bydgoszcz. Informed consent letters for diagnostic tests on admission to the hospital were obtained from the children’s parents.

Antibodies against EBV were detected with the LIAISON^®^ EBV IgM assay (DiaSorin, Saluggia, Italy) using chemiluminescence immunoassay (CLIA) technology. The test was performed on a LIAISON^®^ XL analyser. Isolation of EBV DNA genetic material was performed using the Maxwell RSC Viral Total Nucleic Acid Purification Kit. A real-time polymerase chain reaction (PCR) method was employed to quantify EBV DNA viral load using TaqMan probes according to the manufacturer’s protocol, using a pair of primers with sequences: ForEBV 5′-GGGCTCTGGAGGCACCTA-3′ and RevEBV 5′-CCACCCCA GTCCCGTC-3′. A 99 bp fragment of the non-coding region of the EBV genome (nucleotides 13,640–13,739) was amplified by primers using a 16 bp molecular probe: Probe 5′-TCGAGGCAGGCTTACA-3′. Sensitivity levels of 16 copies/mL were applied. ALT and GTP activities were determined according to the manufacturer’s protocols using a standard enzyme-colourimetric assay (COBAS INTEGRA 400/800, Roche, Grenzacherstrasse, Switzerland). CRP concentration was determined by an immunoturbidimetric method using mouse antibodies against human CRP, according to the manufacturer’s protocol (COBAS INTEGRA 400/800, Roche). IL-6 and TNF-α concentrations were assessed by an immunoenzymatic method based on a commercial enzyme-linked immunosorbent assay (ELISA) kit (Cusabio Biotech, Houston, TX, USA) according to the manufacturer’s protocol using an Etimax 3000 analyser (DiaSorin). Assessment of s-ICAM-1 concentration was performed by an immunoenzymatic method based on a commercial ELISA kit (Wuhan Fine Biotech, Wuhan, China) according to the manufacturer’s protocol. The assay was performed using an Etimax 3000 automatic analyser (DiaSorin).

### Statistical Analysis

The values of the study parameters were measured as categorical and quantitative variables. Categorical variables were described using counts and percentages (%), while quantitative variables were described using medians and 25th and 75th percentiles (Q1–Q3), the distribution of most of the analysed variables did not conform to a normal distribution. Differences in the distributions of categorical variables were assessed using Pearson’s chi-squared test or Fisher’s exact test in the case of small group sizes. For comparisons of quantitative variables, non-parametric tests for independent groups, such as the Mann–Whitney U test for two groups or the Kruskal–Wallis test for the simultaneous comparison of more than two groups. A value of *p* < 0.05 was selected as the significance level for statistical tests.

## 3. Results

**Figure 1:**
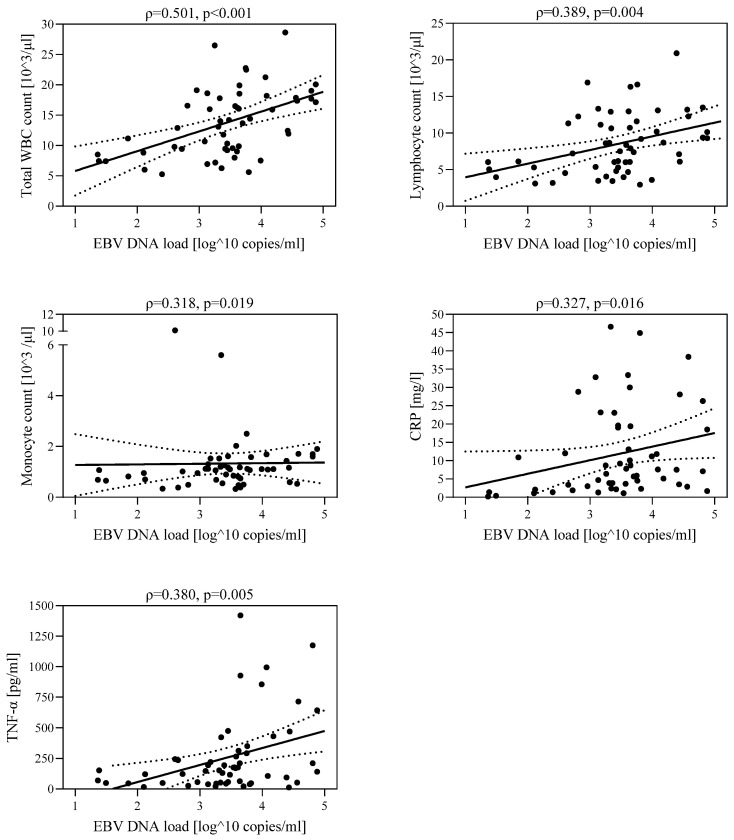
Correlations between EBV DNA viral load and leukocyte count, lymphocyte count, monocyte count, CRP concentration, and TNF-α concentration in the group of children with hepatological complications (IA+IB): IA—group with increased ALT and GGTP activity, IB—group with increased ALT and normal GGTP activity.

In the study group of 54 children diagnosed with hepatitis, the relationship between EBV DNA viral load and the number of leukocytes (ρ = 0.501, *p* < 0.001), number of lymphocytes (ρ = 0.389, *p* = 0.004), number of monocytes (ρ = 0.318, *p* = 0.019), CRP level (ρ = 0.327, *p* = 0.016), and TNF-α concentration (ρ = 0.380, *p* = 0.005) was assessed (Figure 1). A higher viral load was associated with increased leukocyte count, lymphocyte count, monocyte count, CRP concentration, and TNF-α concentration.

The analyses revealed that IL-6, TNF-α, and s-ICAM-1 levels were higher in EBV-infected children than in the uninfected patients (9.6 vs. 3.7 pg/mL, *p* = 0.006; 121.0 vs. 42.0 pg/mL, *p* = 0.001; and 10.0 vs. 5.8 ng/mL, *p* = 0.074, respectively) (Table 1).

**Figure 2:**
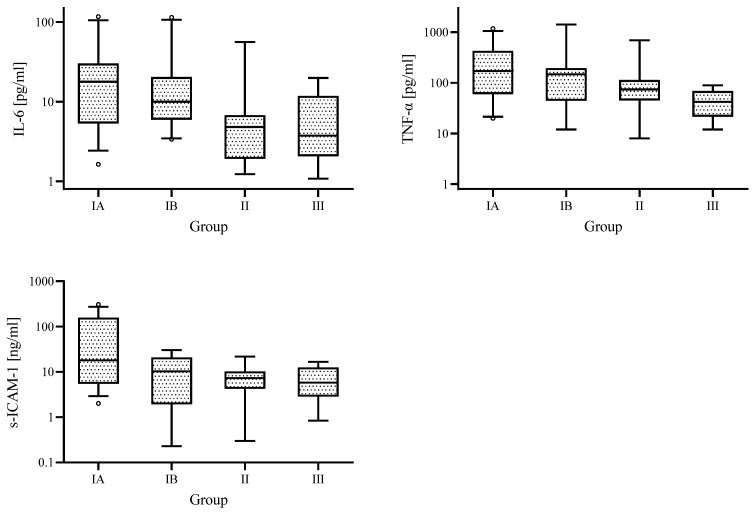
Medians of IL-6 and TNF-α, s-ICAM-1 concentrations for the different analysed groups; IA—group with increased ALT and GGTP activity, IB—group with increased ALT and normal GGTP activity, II—control group with normal ALT and GGTP activity, and III—comparative uninfected group.

In EBV-infected children, concentrations of IL-6, TNF-α, and s-ICAM-1 were the highest in Group IA and the lowest in Group II (Figure 2).

The median concentrations of the selected laboratory parameters are shown in Table 2.

There were statistically significantly higher IL-6 concentrations in Group IA and Group IB than in Group II (median 17.8 vs. 4.8 pg/mL, *p* = 0.005; 8.1 vs. 4.8 pg/mL, *p* = 0.008, respectively) and statistically significantly higher TNF-α and s-ICAM—1 concentrations in Group IA than in Group II (170.0 vs. 74.0 pg/mL, *p* = 0.031; 18.0 vs. 7.2 ng/mL, *p* = 0.021, respectively). ALT activity in Group IA and IB was statistically significantly higher than in Group II (186.0 vs. 22.0 IU/L, *p* < 0.001; 58.0 vs. 22.0 IU/L, *p* < 0.001, respectively).

In groups IA and IB, GGTP activity was statistically significantly higher than in Group II (median 93.0 vs. 11.5 IU/L, *p* < 0.001; 21.0 vs. 11.5 IU/L, *p* = 0.002). In Group IB, GGTP activity was still within the normal range for age and was not clinically significant.

**Figure 3:**
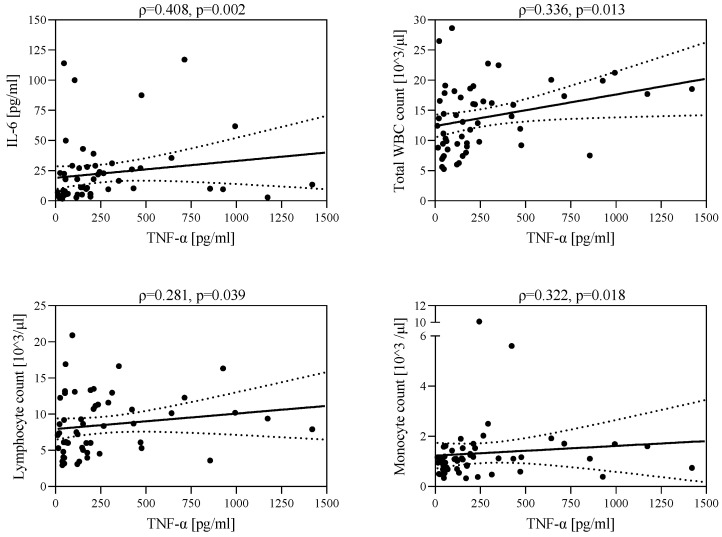
Correlations between TNF-α level and IL-6 level, leukocyte count, lymphocyte count, and monocyte count.

In the group of patients with hepatitis in the course of EBV infection, there was a negative correlation between s-ICAM-1 level and IL-6 level (ρ = −0.464, *p* < 0.001) and a positive correlation between TNF-α concentration and the number of leukocytes (ρ = 0.336, *p* = 0.013), lymphocytes (ρ = 0.281, *p* = 0.039), monocytes (ρ = 0.322, *p* = 0.018), and IL-6 concentration (ρ = 0.408, *p* = 0.002) (Figure 3). A higher TNF-α concentration was associated with increased leukocyte count, lymphocyte count, monocyte count, and IL-6 concentration.

**Figure 4:**
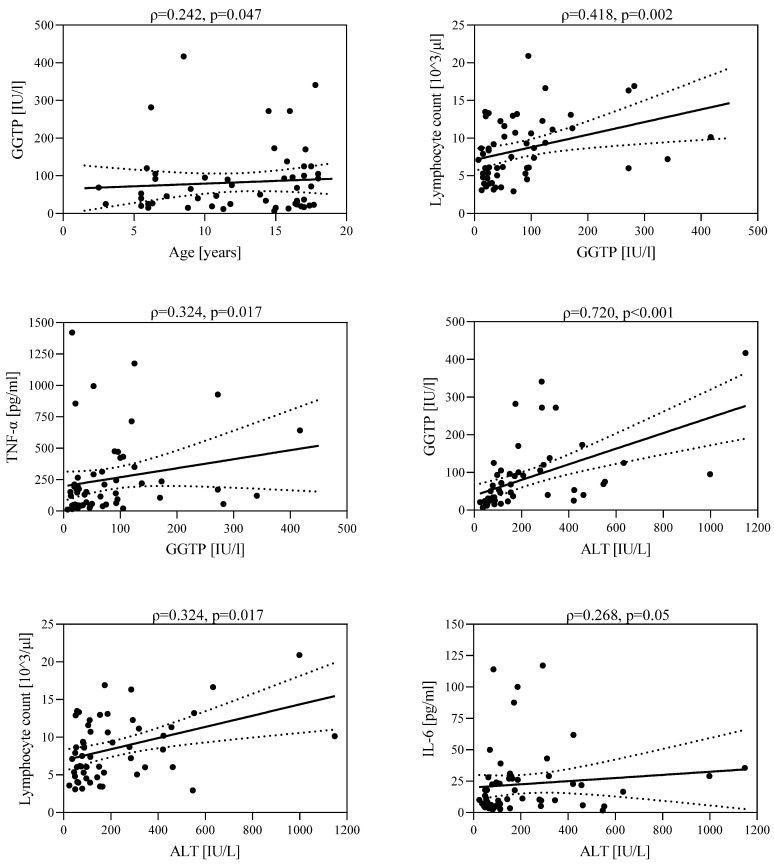
Correlations between GGTP activity and age; lymphocyte count and TNF-α concentration; and between ALT activity and GGTP activity; lymphocyte count and IL-6 concentration.

In the group of children with hepatitis, there was a positive correlation between GGTP activity and children’s age (ρ = 0.242, *p* = 0.047), lymphocyte count (ρ = 0.418, *p* = 0.002), and TNF-α concentration (ρ = 0.324, *p* = 0.017). A higher GGTP activity was observed in older children. As GGTP activity increased, an increase in lymphocyte count and TNF-α concentration was observed. In addition, a positive statistically significant correlation was found between ALT activity and GGTP activity (ρ = 0.720, *p* < 0.001), lymphocyte count (ρ = 0.324, *p* = 0.017), and IL-6 concentration (ρ = 0.268, *p* = 0.05). As ALT activity increased, an increase in GGTP activity, lymphocyte count, and IL-6 concentration was observed (Figure 4).

## 4. Discussion

EBV infections in children are usually asymptomatic. Infection in adolescents and adults can lead to the development of infectious mononucleosis, usually presenting with a triad of symptoms: fever, cervical lymphadenopathy, and pharyngitis [10,11]. In the acute phase of primary EBV infections, an increase in liver enzymes is often observed. Mild to moderate increases in ALT affect 80–90% of patients. Severe cholestatic hepatitis occurs in only 5% of cases [12,13]. Due to cholestatic liver damage, bile acid transport is impaired. The disruption of the blood supply to liver cells as a consequence of cholestasis significantly contributes to the inhibition of the activity of protein biliary transporters, which may be reflected in elevated levels of TNF-α, IL-1β (interleukin-1β), and IL-6 [14]. Hornef et al. showed statistically significantly higher levels of TNF-α and IL-6 in patients with infectious mononucleosis under LPS stimulation than in the controls [15]. In our study, we confirmed that in the group of EBV-infected children without hepatological complications as well as those with hepatitis and concomitant biliary pole damage, the levels of IL-6 and TNF-α were significantly higher than in patients not infected with EBV. Furthermore, a positive correlation between these parameters was shown in this group of children. This finding supports the thesis of endothelial cell activation and the associated increased expression of pro-inflammatory cytokines due to hepatocyte damage following EBV infection [14,16,17].

IL-6 is secreted mainly by immune cells, i.e., B and T lymphocytes, among others. It stimulates, among other things, the secretion of acute-phase proteins in hepatocytes and influences the differentiation of B lymphocytes and the activation of T lymphocytes. Therefore, an increase in the expression of IL-6 levels may accelerate EBV replication and the expansion of EBV-infected B lymphocytes [16,18]. An early response to infection and tissue damage is the acute-phase response, characterised by the rapid production of many proteins. The majority of acute-phase proteins are produced in the liver, and the main inducer of their synthesis is IL-6, which activates hepatocytes. In some studies, IL-6 has been shown to be a more relevant prognostic factor in cases of severe systemic inflammation in patients with concomitant severe liver dysfunction than leukocyte count or CRP level [19]. There are reports in which increased CRP levels have been indicated to be associated with increased mortality in patients with cirrhosis [20,21]. A study by Remmler et al. indicated that not only CRP concentration, but also leukocyte count and IL-6 concentration can be considered predictive of death in patients with cirrhotic liver failure [21]. The study also indicated that IL-6 concentration has the greatest significance, comparable to the Model of End-Stage Liver Disease (MELD) scale, in monitoring the degree of liver failure. Our study showed significantly higher IL-6 concentrations in both EBV-infected children with hepatitis and those with hepatocyte and biliary pole damage. In the group of patients with hepatological complications during the course of EBV infection, a positive correlation between ALT activity and IL-6 concentration was also demonstrated. Our own data confirm the possibility of using IL-6 as a predictor of hepatocyte damage in the course of EBV infection.

In the course of hepatitis, damage to hepatocytes results, among other things, from impaired blood supply, to which endothelial damage contributes. As a result of endothelial damage, there is not only an increased expression of IL-6, but also TNF-α. In our study, a positive correlation between EBV DNA viral load and leukocyte count, CRP and TNF-α level, as well as between TNF-α level and IL-6 level, was demonstrated both in the whole EBV-infected group and in a subgroup of children with hepatological complications in the course of EBV infection. Endothelial damage also leads to an increased leukocyte adhesion and an increased secretion of s-ICAM-1 [22]. The importance of s-ICAM-1 in patients with extrahepatic and intrahepatic cholestasis has been described in numerous studies, which confirmed statistically significant correlations between s-ICAM-1 levels and GTTP activity, alkaline phosfatase (ALP) activity, aspartate aminotransferase (AST) activity, or bilirubin level [5,23]. Tomasiewicz et al. confirmed a statistically significant increase in s-ICAM-1 levels in patients with infectious mononucleosis compared to healthy controls [24]. Liver damage due to cholestasis includes the lytic necrosis of hepatocytes, the activation of phagocytosis and Kupffer cells, and the recruitment of neutrophils and mononuclear cells in the parenchyma. To participate in the elimination of necrotic hepatocytes, neutrophils must migrate through hepatic sinusoidal cells. The adhesion particle increases the adhesion of leukocytes and the migration of lymphocytes with secretory abilities. An increased secretion of TNF-α, IL-1, and IL-6 stimulates the intensification of the secretion of the s-ICAM-1. Therefore, hepatitis accompanied by cholestasis increases de novo ICAM-1 expression within sinusoidal endothelial cells and Kupffer cell [6,25].

Our study did not show significantly higher s-ICAM-1 concentrations in EBV-infected patients compared to uninfected patients. However, it was confirmed that the concentration of the adhesion molecule was the highest in the group of EBV-infected children with concomitant hepatitis and biliary pole injuries compared to patients without hepatological complications. Our own data, as well as the studies by Hornef and Tomasiewicz [15,24], suggest an effect of EBV infection on TNF-α, IL-6, and s-ICAM-1 concentrations and the possibility of their use as predictive factors for the development of hepatological complications among EBV-infected children.

The EBV DNA viral load is an important predictor of the severity of the course of infection in immunocompetent patients. It correlates with disease severity and is also used to assess response to treatment [26,27,28,29]. In immunocompetent patients, an EBV DNA viral load assessment is not currently recommended for the routine diagnosis of EBV infection. A pilot in-house study enrolling 36 children with serologically and molecularly confirmed EBV infection, as well as the current study enrolling 68 children, showed a statistically significant correlation between EBV DNA viral load and CRP level, TNF-α concentration, leukocyte count, and monocyte count [30]. A group of 54 children with hepatological complications in the course of EBV infection were analysed in detail, in which correlations among ALT activity, GGTP activity, the duration of symptoms, selected elements of the protein–cell system, and EBV DNA viral load levels were analysed. It was shown that patients with a higher viral load were characterised by higher leucocytosis, lymphocytosis, CRP, and TNF-α levels. These data are consistent with the results obtained in a pilot study in which 26 patients with hepatological complications were selected from a group of 36 infected children. In this group, statistically significant correlations were also obtained between EBV DNA viral load and CRP and TNF-α concentrations. In both studies, there was no correlation between EBV DNA viral load level and GGTP activity. Similar to the study by Banko, Bauer, Pitetti, and Kimura, no correlation between EBV DNA viral load level and ALT activity was confirmed in our own pilot study [31,32,33,34].

In our study of a group of EBV-infected children with hepatitis, we confirmed that an increased GGTP activity correlates with increased lymphocyte counts and TNF-α concentrations. These observations suggest that the risk of hepatological complications increases with increasing lymphocytosis and TNF-α concentration. These data are consistent with the pathomechanism of cholestatic hepatitis presented by Shaklim-Zemer [13]. According to this theory, EBV does not directly destroy liver cells or the bile duct epithelium. Virus-infected CD8 + T lymphocytes accumulate in the liver and activate pro-inflammatory cytokines such as interferon γ or TNF-α, which directly damage hepatocytes. Furthermore, in the group of 17 children with cholestatic hepatitis induced by EBV infection analysed by Shaklima-Zemer, five patients were under the age of four. This analysis, as well as our own study, which showed that among 54 patients with hepatological complications from EBV infection, only two patients were <five years of age, suggests a higher incidence of hepatological complications of infectious mononucleosis in a group of older children. The rarer occurrence of hepatological disorders in the youngest group of patients, in which the rate of false-negative results for heterophilic or specific antibodies directed against the capsid antigen is the highest, confirms that EBV DNA viral load determination can be helpful in the differential diagnosis in this age group [35,36]. In their study, Shi et al. emphasised the importance of EBV DNA viral load determination, especially in patients under seven years of age manifesting symptoms that may suggest a disease entity induced by EBV infection [37].

## 5. Conclusions

It appears that an increase in IL-6, TNF-α, and s-ICAM-1 in the course of EBV infection may indicate the risk of hepatitis with concomitant biliary pole damage. The level of EBV DNA viral load may be helpful in assessing the indications for hospitalisation in a group of patients with elevated inflammatory exponents.

## Figures and Tables

**Table 1:** Concentrations of IL-6, TNF-α, and s-ICAM-1 in infected and uninfected children (Group III) EBV.

	Infected with EBV N = 68	III N = 13	*p*-Value
	Median (Q1–Q3)	Median (Q1–Q3)	
IL-6 (pg/mL)	9.6 (4.9–26.5)	3.7 (2.5–6.3)	0.006
TNF-α (pg/mL)	121.0 (52.0–255.0)	42.0 (22.0–57.0)	0.001
s-ICAM-1 (ng/mL)	10.0 (4.6–26.0)	5.8 (3.8–11.3)	0.074

**Table 2:** Median values (Q1–Q3) for the parameters analysed in Group IA (*n* = 33), Group IB (*n* = 21), and Group II (*n* = 14); IA—group with increased ALT and GGTP activity, IB—group with increased ALT and normal GGTP activity, and II—control group with normal ALT and GGTP activity.

	Group IA	Group IB	Group II	*p*-Value IA vs. II	*p*-Value IB vs. II
Age (years)	14.5 (6.5–17.0)	14.3 (8.8–16.5)	8.5 (5.9–14.5)	0.087	0.089
Duration of hospitalisation (days)	5.0 (4.0–8.0)	4.0 (3.0–5.0)	5.0 (4.0–6.0)	0.860	0.229
Duration of symptoms (days)	6.0 (4.0–11.0)	7.0 (3.0–10.0)	4.5 (3.0–8.0)	0.304	0.302
EBV DNA (copies/mL)	4341.0 (1778.0–12,233.0)	2134.0 (653.0–4090.0)	1797.0 (1306.0–7670.0)	0.329	0.544
EBV DNA (copies/mL, log10)	3.6 (3.2–4.1)	3.3 (2.8–3.6)	3.2 (3.1–3.9)	0.329	0.544
ALT (IU/L)	186.0 (113.0–345.0)	58.0 (49.0–85.0)	22.0 (13.0–26.0)	<0.001	<0.001
WBC (10^3^/µL)	15.9 (9.8–18.2)	11.2 (8.8–16.5)	11.7 (9.2–14.9)	0.209	0.920
Lymphocytes (10^3^/µL)	9.3 (6.0–11.6)	6.0 (4.6–8.6)	6.7 (3.8–10.1)	0.125	0.814
Monocytes (10^3^/µL)	1.1 (0.6–1.6)	1.1 (0.8–1.3)	0.9 (0.7–1.3)	0.625	0.906
GGTP (IU/L)	93.0 (65.0–125.0)	21.0 (15.0–26.0)	11.5 (10.0–16.0)	<0.001	0.002
CRP (mg/L)	7.6 (3.7–18.5)	6.4 (2.1–19.4)	12.1 (4.9–19.0)	0.306	0.219
IL-6 (pg/mL)	17.8 (5.5–29.1)	8.1 (6.1–17.9)	4.8 (1.9–6.4)	0.005	0.008
TNF-α (pg/mL)	170.0 (63.0–423.0)	147.0 (45.0–194.0)	74.0 (52.0–105.0)	0.031	0.429
s-ICAM-1 (ng/mL)	18.0 (6.1–157.0)	10.3 (2.0–20.4)	7.2 (4.8–9.8)	0.021	0.400

## Data Availability

Data available on request due to restrictions, e.g., privacy or ethical. The data presented in this study are available on request from the corresponding author.
